# Why Radiological Confirmation is Necessary: A Case of Misplaced Temporary Dialysis Catheter into the Axillary Vein

**DOI:** 10.7759/cureus.83623

**Published:** 2025-05-07

**Authors:** Hamza Arif, Hassaan Iftikhar, Maryam Saleem

**Affiliations:** 1 Nephrology, Ohio Valley Nephrology Associates, Owensboro, USA; 2 Nephrology, Washington University School of Medicine, Saint Louis, USA; 3 Internal Medicine, Saint Francis Medical Center, Trenton, USA; 4 Internal Medicine, Waterbury Hospital, Waterbury, USA

**Keywords:** central venous catheter (cvc), complications of cvc placement, dialysis catheter, end stage renal disease (esrd), hd (hemodialysis), hemodialysis access, malposition of cvc, temporary dialysis catheter, tunneled dialysis catheter

## Abstract

A 67-year-old male with a history of end-stage kidney disease requiring in-center hemodialysis (HD) three days a week via left brachiocephalic graft presented with altered mental status. The hospital course was complicated by graft thrombosis. As the patient could not undergo thrombectomy immediately, he underwent temporary HD catheter using ultrasound-guided right internal jugular vein (IJV) cannulation. Post-procedure chest X-ray to confirm this access indicated that this central venous catheter (CVC) was misplaced, with its tip placed into the right axillary vein. This access was not used for HD, and he eventually underwent thrombectomy of the graft and angioplasty of the graft stenosis. The patient later underwent tunneled HD catheter placement under fluoroscopy at the time of thrombectomy, which revealed a torturous superior vena cava, which may indicate why the temporary HD catheter entered the subclavian vein, eventually with its tip at the right axillary vein. This report highlights the crucial importance of radiological confirmation of any CVC placement, especially HD catheters. HD should not be performed until the location of the dialysis catheter has been verified.

## Introduction

Complications related to central venous catheter (CVC) placements are well known and include pneumothorax, infection, thrombosis, and misplaced catheter location [1]. These concerns are particularly enhanced in patients who undergo temporary hemodialysis (HD) catheters, given their larger size. The preferred access for patients on HD is an arteriovenous fistula or graft, but access to these locations can be complicated by stenosis, vein thrombosis, and occlusion that can necessitate the placement of HD catheters [2]. Although CVC misplacement has been previously described, its incidence is lower when ultrasound guidance is used to place them [3]. Most commonly, these misplaced catheters are in the azygous veins or subclavian veins. However, we describe a case of a misplaced HD catheter into the right axillary vein.

## Case presentation

**Figure 1:**
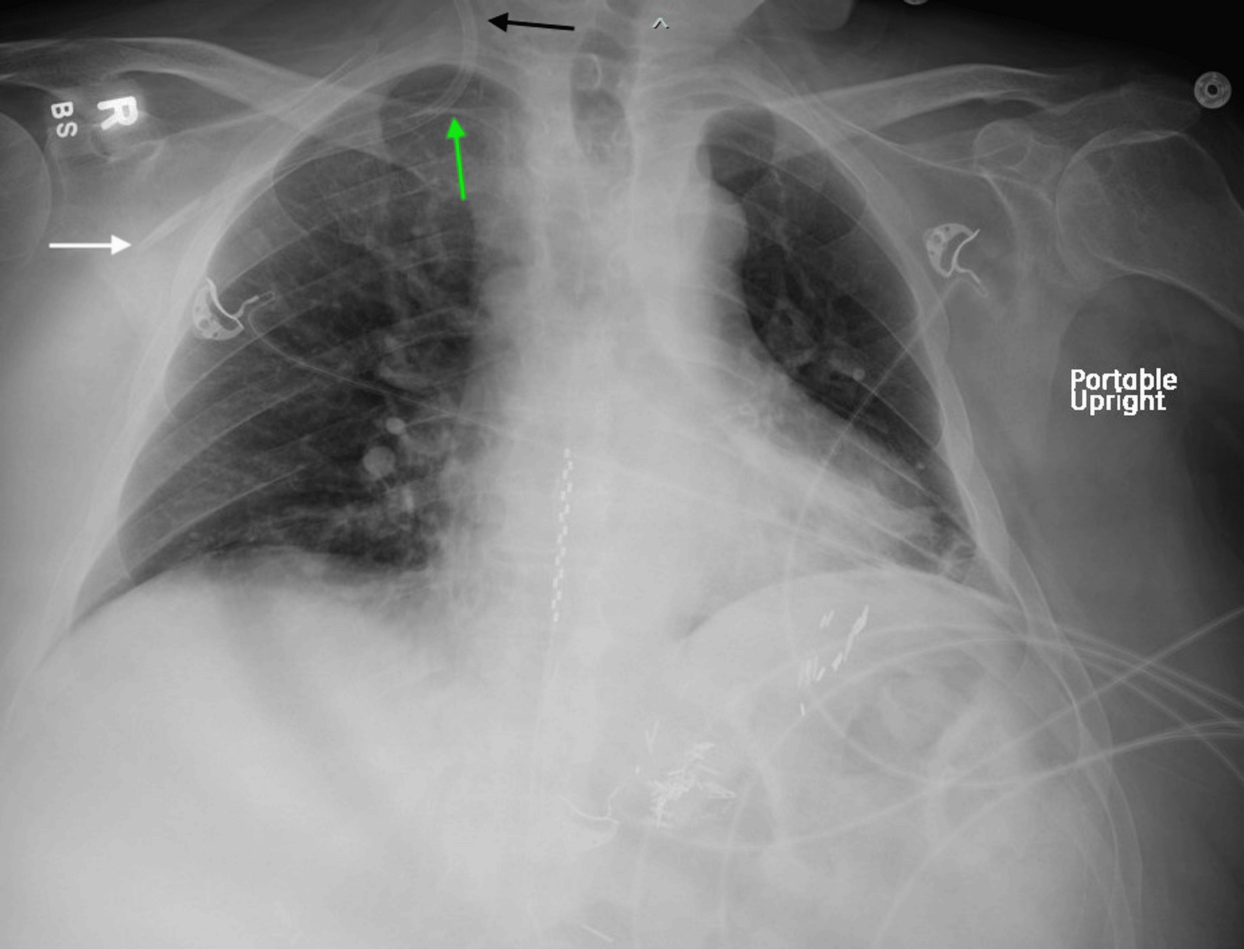
Portable chest X-ray demonstrating a right IJV temporary HD catheter extending laterally into the subclavian vein, with its tip terminating in the right axillary vein Black arrow: catheter in the IJV. Green arrow: catheter deviating into the subclavian vein instead of continuing toward the superior vena cava. White arrow: catheter tip located in the right axillary vein HD: hemodialysis; IJV: internal jugular vein

A 67-year-old male with a history of end-stage kidney disease requiring in-center HD three days a week via left brachiocephalic graft presented to the hospital with altered mental status. He also had chronic pain and had a prior hospitalization with similar complaints in the setting of baclofen use. On admission, he was hypotensive, requiring vasopressors, and was admitted to the ICU. His laboratory work was unremarkable, and he underwent HD using his left brachiocephalic graft; however, this was complicated by access thrombosis. Due to the surgeon’s unavailability for thrombectomy and angioplasty, the decision was made to place a temporary HD catheter through ultrasound-guided right internal jugular vein (IJV) cannulation. Post-procedure chest X-ray to confirm this access indicated that this CVC was misplaced, with its tip placed into the right axillary vein (Figure 1). This access was subsequently not utilized for HD, and the catheter was removed. He eventually underwent thrombectomy of the graft and angioplasty of the graft stenosis, and finally underwent successful HD. His clinical status improved over the next few days, and he was eventually discharged.

## Discussion

Axillary vein is a continuation of the basilic vein and is lateral to the subclavian vein. While misplaced CVCs are not uncommon [4], this usually involves placement into the azygous veins and subclavian veins. Insertion of a CVC into an axillary vein is described less often [5] and is usually due to anatomical variation or operator error [3]. HD performed via the dialysis access requires high flow, typically using blood flow rates (BFR) of 300-400 ml/min. This is usually not feasible in an inappropriate location such as the axillary vein, although a prior study did demonstrate success in achieving BFR of at least 300 ml/min in axillary veins [6]. Furthermore, axillary vein catheter placement can place patients at risk of complications such as hematoma formation and nerve injury [7] along with other well-known risks associated with CVCs, such as thrombosis or infection. A prior report described the placement of a CVC into the right axillary vein, leading to thrombosis of the axillary vein after it had been in place for six days [8]. This further highlights that the catheter tip position is crucial in HD, as the catheter needs to be in a central location to ensure high blood flow rates.

As described above, the patient’s temporary HD catheter with its tip in the right axillary vein was not utilized for HD. He eventually underwent thrombectomy of the graft and angioplasty of the graft stenosis, and that access was successfully cannulated for HD. However, he also underwent tunneled HD catheter placement under fluoroscopy at the time of thrombectomy, as there was concern for extensive disease in the graft. Fluoroscopy revealed a tortuous superior vena cava, which may indicate why the temporary HD catheter had entered the subclavian vein, eventually with its tip at the right axillary vein. Notably, during the placement of the temporary HD catheter, no difficulty was encountered when advancing the wire through the right IJV, despite ultrasound guidance, suggesting that this tortuosity was subtle.

Proper confirmation of catheter position following CVC insertion is crucial in avoiding the risks associated with misplacement [4]. Although ultrasound guidance significantly reduces the risk of malposition during catheter insertion and can help to ensure correct placement in central veins, that is not always the case, as described in our case report.

## Conclusions

Misplacement of a temporary HD catheter into the right axillary vein represents a rare but significant deviation from the ideal catheter placement scenarios. This report highlights the critical importance of radiological confirmation of any CVC, especially HD catheters, prior to their use, even when placed under ultrasound guidance. HD should not be performed until the location of the dialysis catheter has been verified.
